# Effects of Neuromuscular Electrical Stimulation for Quadriceps Muscle Thickness and Lower Extremity Motor Score in Individuals with Subacute Incomplete Cervical Spinal Cord Injury: A Randomized Controlled Trial

**DOI:** 10.1298/ptr.E10291

**Published:** 2024-10-04

**Authors:** Yusuke MOROOKA, Yosuke KUNISAWA, Yuya OKUBO, Yasuyuki TAKAKURA

**Affiliations:** 1 Department of Physical Therapy, Faculty of Health and Medical Care, Saitama Medical University, Japan; 2 Department of Rehabilitation, Saitama Medical Center, Japan

**Keywords:** Muscular atrophy, Incomplete cervical spinal cord injury, Neuromuscular electrical stimulation

## Abstract

Objective: In this study, we aimed to determine the effects of 2-week neuromuscular electrical stimulation (NMES) on quadriceps muscle atrophy and lower extremity motor score in individuals with subacute incomplete cervical spinal cord injury (SCI). Methods: This stratified randomized controlled trial, conducted in the advanced critical care center of a university hospital, comprised 49 individuals with American Spinal Injury Association (ASIA) impairment scale grade C and D incomplete cervical SCI. The participants were stratified based on the ASIA impairment scale grade and randomly assigned to the control (n = 25) or NMES (n = 24) group. The control group participants received only conventional rehabilitation; the NMES group participants received conventional rehabilitation plus NMES in the quadriceps muscles of both lower limbs. The primary endpoints were quadriceps muscle thickness and L3 ASIA lower extremity motor score (L3 motor score), measured at the study’s initiation and after 2 weeks. Results: The quadriceps muscle thickness changes on the stronger and weaker sides were –14.2% ± 11.3% and –15.1% ± 13.8%, respectively, in the NMES group and –25.7% ± 16.8% and –26.0% ± 13.3%, respectively, in the control group, indicating significantly lesser reduction on both sides in the NMES group (*p* <0.05). The L3 motor scores on the stronger and weaker sides were 0.8 ± 1.2 and 1.3 ± 1.4 (NMES group) and 0.4 ± 0.8 and 0.4 ± 0.8 (control group), respectively, indicating significant improvement only on the weaker side (*p* <0.05). Conclusions: For subacute incomplete cervical SCI, 2 weeks of NMES reduces quadriceps muscle atrophy and improves the L3 motor score values on the weaker side compared with standard treatment.

## Introduction

In Japan, 75% of spinal cord injuries (SCI) affect the cervical spine^1)^, with 90% being incomplete injuries^2)^. As falls from standing height in the aging population become more frequent, cases of incomplete cervical SCI are expected to increase. Internationally, the proportion of cervical SCI ranges from 22% to 55% in various regions^1)^. In addition, studies in Western countries report that approximately 55% to 60% are incomplete^3^^,^^4)^. These differences highlight the higher prevalence of incomplete cervical SCI in Japan compared to other regions. The goal of rehabilitation in the acute and subacute phases after incomplete cervical SCI is to limit complications related to respiratory impairments^5^^,^^6)^ and provide opportunities for functional recovery from motor paralysis^7)^. Preventing disused muscle atrophy is important for motor improvement and limiting secondary complications, such as decubitus ulcer, fractures, and deep vein thrombosis^8)^.

Lower limb muscle disuse atrophy following SCI is well described in the chronic and subacute stages^9–12)^. Measures of disuse atrophy occurring following immobility from critical illness or acute stroke demonstrate changes in rectus femoris and vastus medialis thickness 2–3 weeks after injury^13^^,^^14)^. Therefore, muscle atrophy is expected to occur soon after injury in individuals with incomplete cervical SCI. Evidence supports the use of neuromuscular electrical stimulation (NMES) to target disuse atrophy following SCI. NMES induces muscle contraction by electrically stimulating the innervating nerve or the muscle’s membrane potential^15)^. A systematic review^16)^ of early exercise interventions targeting muscle atrophy in subacute SCI identified four randomized controlled trials evaluating NMES. These trials evaluated young participants for 2–10 weeks^17^^–^^20)^. The use of NMES in the immediate post-injury stage has yet to be extensively evaluated, and this is the basis of our research question.

In this study, we aimed to compare the thickness and lower extremity motor score of the quadriceps muscle after NMES for 2 weeks post-injury to clarify the effects of short-term NMES in preventing muscle atrophy in individuals with subacute (3–20 days after injury) incomplete cervical SCI. We hypothesized that compared with physical therapy (PT) alone, NMES combined with PT would prevent muscle atrophy and improve lower extremity motor scores in individuals with incomplete SCI.

## Methods

### Study design and participants

The study participants comprised consecutively enrolled individuals aged <90 years with cervical SCI classified as American Spinal Injury Association (ASIA) Impairment Scale grades C and D who were admitted to an advanced critical care center of a university hospital within 48 h of injury between February 2021 and March 2022. ASIA Impairment Scale (AIS) grade C is less than half of the key muscle functions below the single neurological level of injury (NLI) having a muscle grade ≥3. AIS grade D is at least half (or more) of the key muscle functions below the single NLI having a muscle grade ≥3^21)^. The exclusion criteria were as follows: presence of cerebrovascular, orthopedic, or cardiac disease, on dialysis, trauma or fracture of the pelvis or lower extremities, and presence of dermatitis, malignancy that limits NMES use. All participants were tested for COVID-19 and found to be negative. The participants were randomized using computer-generated random numbers, stratified 1:1 according to the AIS grade, and assigned to the control or NMES group. The researcher recruited the participants by blinding them to the order of grouping to minimize selection bias.

The sample size required for this study was calculated using R4.0.2 (CRAN, Freeware) and R Commander 2.7.0 (R Software for Statistical Computing, Vienna, Austria). Based on the results of a previous study^22)^, with an effect size of 0.8, a significance level of 0.05, and a power of 0.8, a sample size of 52 participants in the two groups was required. In acute care hospitals, we anticipated that the number of participants who dropped out of the study would increase because participants were suddenly transferred to hospitals due to the shortened length of stay. Therefore, we aimed to include 52 participants in the final analysis.

### Intervention

The control group received only PT, and the NMES group received PT combined with NMES. In the NMES group, NMES was applied to the quadriceps muscles of both lower extremities for 2 weeks (5 days/week)^22)^ starting at the first measurement date. NMES was not conducted on the remaining 2 days per week. Self-adhesive electrodes (90 × 50 mm) manufactured by Axelgaard (Fallbrook, CA, USA) were applied to the quadriceps muscles of both lower limbs^23)^. The stimulator was an ESPURGE (Ito, Tokyo, Japan), a low-frequency therapy device. The settings were as follows: frequency, 50 Hz; pulse duration, 300 μs; the intensity that could induce visible contraction tolerable by the participants (15–40 mA); and duty cycle, 1:1 for 6 s^22^^,^^24)^. The stimulation duration was 40 min. A previous study^22)^, which demonstrated the efficacy of similar NMES protocols in patients with acute stroke, has shown significantly fewer quadriceps muscle thickness reductions using comparable NMES settings for 2 weeks.

All participants received 40–60 min of PT 5 times weekly as part of standard care. PT interventions included early mobilization, lower limb extension resistance exercises, basic movement practice, and gait practice using a walking aid to improve the participant’s independence. There was no difference in the percentage of gait practices performed in the two groups. The physician determined the timing for the initiation of rehabilitation based on each participant’s condition. Daily occupational and speech therapies were prescribed, as required. There was no difference in the amount of occupational and speech therapy provided to the two groups.

### Measurements

The following data were collected from the medical records: age, sex, body mass index, AIS, injury history, methods of treatment, number of days from injury to the start of PT, number of days from injury to the start of measurement, serum albumin level, and C-reactive protein (CRP). The primary outcome measures included the thicknesses of the rectus femoris and vastus medialis muscles, combined and reported as the total quadriceps muscle thickness, and the L3 ASIA lower extremity motor score (L3 motor score). The L3 motor score is a component of the international standards for the neurological classification of SCI, a standardized examination used to assess neurological function and classify SCI^21)^. In the NMES group, continuous monitoring for adverse events related to NMES was conducted throughout the trial.

**Fig. 1.**
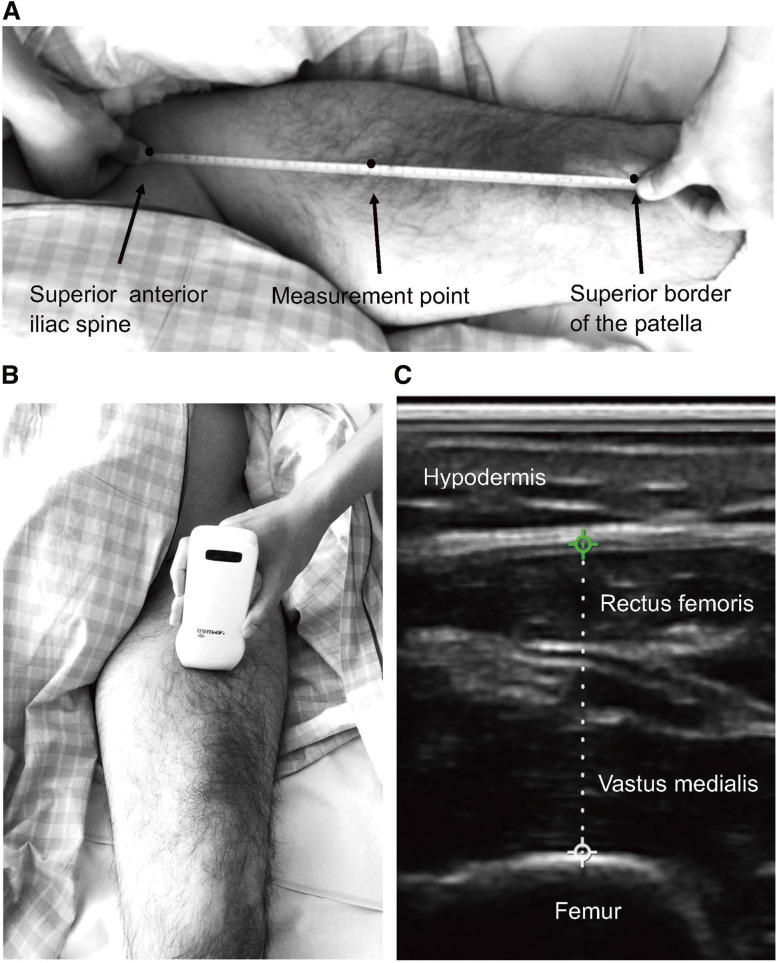
Ultrasound image of representative quadriceps muscle thickness with probe placement and measurement method (A) The measurement site. (B) The location of the probe. (C) A digital tape measure built into the US device was used to measure the thickness of the rectus femoris and vastus medialis muscles located directly above the superior border of the femur. The dotted line indicated by the digital tape measure shows the combined muscle thickness of the rectus femoris and vastus medialis muscles

The primary outcome measures were assessed in all participants after assignment to the control or NMES group on the day informed consent was obtained (within 6 days of injury) and 2 weeks after the first measurement. The thickness of the quadriceps femoris was evaluated using ultrasound (US) by the first author, who was trained. US measurements in individuals with SCI have been used to evaluate the effects of interventions on quadriceps muscle thickness^25)^. The US measurement device (FAMUBO, Seikosha, Tokyo, Japan) was a 10/12-MHz linear probe in B mode. The settings were adjusted according to the participant’s characteristics at a depth of 4–8 cm, at which the femur could be visualized. The measurement site was the midpoint between the superior anterior iliac spine and the superior border of the patella, just above the thigh, with the participant’s lower garments removed. This method has shown excellent reproducibility in previous studies^26)^. In this study, the intraclass correlation coefficient (1,1) for muscle thickness measurements was 0.97, indicating the high reliability of the method. The probe was lightly applied to the skin surface such that the pressure of the probe did not alter the shape of the soft tissue. A digital tape measure built into the US device was used to measure the thickness of the rectus femoris and vastus medialis muscles located directly above the superior border of the femur (Fig. 1). The periosteum and fascia were excluded, and only the parenchyma was measured. One measurement was obtained for each muscle.

The L3 motor score was evaluated on a 6-point scale from 0 to 5 points. The sides with the higher and lower values from the initial measurement were defined as the stronger and weaker, respectively. When both sides had the same value, the side with greater muscle thickness was considered stronger, and the side with lesser muscle thickness was considered weaker. The final measurement also maintained these definitions of the stronger and weaker sides. The first author, a trained, performed all evaluations to reduce measurement bias. In addition, efforts were made to minimize bias by blinding the participants and their physiotherapists to the measurement results.

### Statistical analysis

All data are presented as means ± standard deviations. Statistical tests were performed to compare the control and NMES groups on the measurement items related to the participants’ attributes and physical function using a two-sample t-test, an exact Wilcoxon rank-sum test, Pearson’s chi-squared test, or Fisher’s exact test. Statistical tests performed during the initial and final measurements of quadriceps muscle thickness and L3 motor score in the control and NMES groups were compared using a paired t-test. The change in the initial and final measurements was calculated and compared between the control and NMES groups using a two-sample t-test. This study used a modified intention-to-treat (ITT) analysis^27^^,^^28)^, a broader version of the ITT analysis recommended in the Consolidated Standards of Reporting Trials statement. Participants who experienced events, such as acute discharge or transfer to another hospital within 2 weeks after starting follow-up, and were unable to provide final data for the primary endpoint were excluded from the final analysis. Participants who received NMES despite being in the control group or failed to receive NMES despite being in the NMES group were analyzed as if they belonged to the assigned group. Measurement results were analyzed by statistical analysts blinded to group assignment. The significance level of all statistical methods was set at 5%, and R4.0.2 and R Commander 2.7.0 were used for statistical analyses.

This study was conducted following the Declaration of Helsinki and the Ethical Guidelines for Clinical Research Involving Human Subjects. This study was approved by the Research Ethics Committee of Saitama Medical Center (Approval No: 2474) and registered at the University Hospital Medical Information Network-Clinical Trials Registry (UMIN-CTR-41303). Oral and written informed consent was provided by all participants before data collection.

## Results

### Study participants

**Fig. 2.**
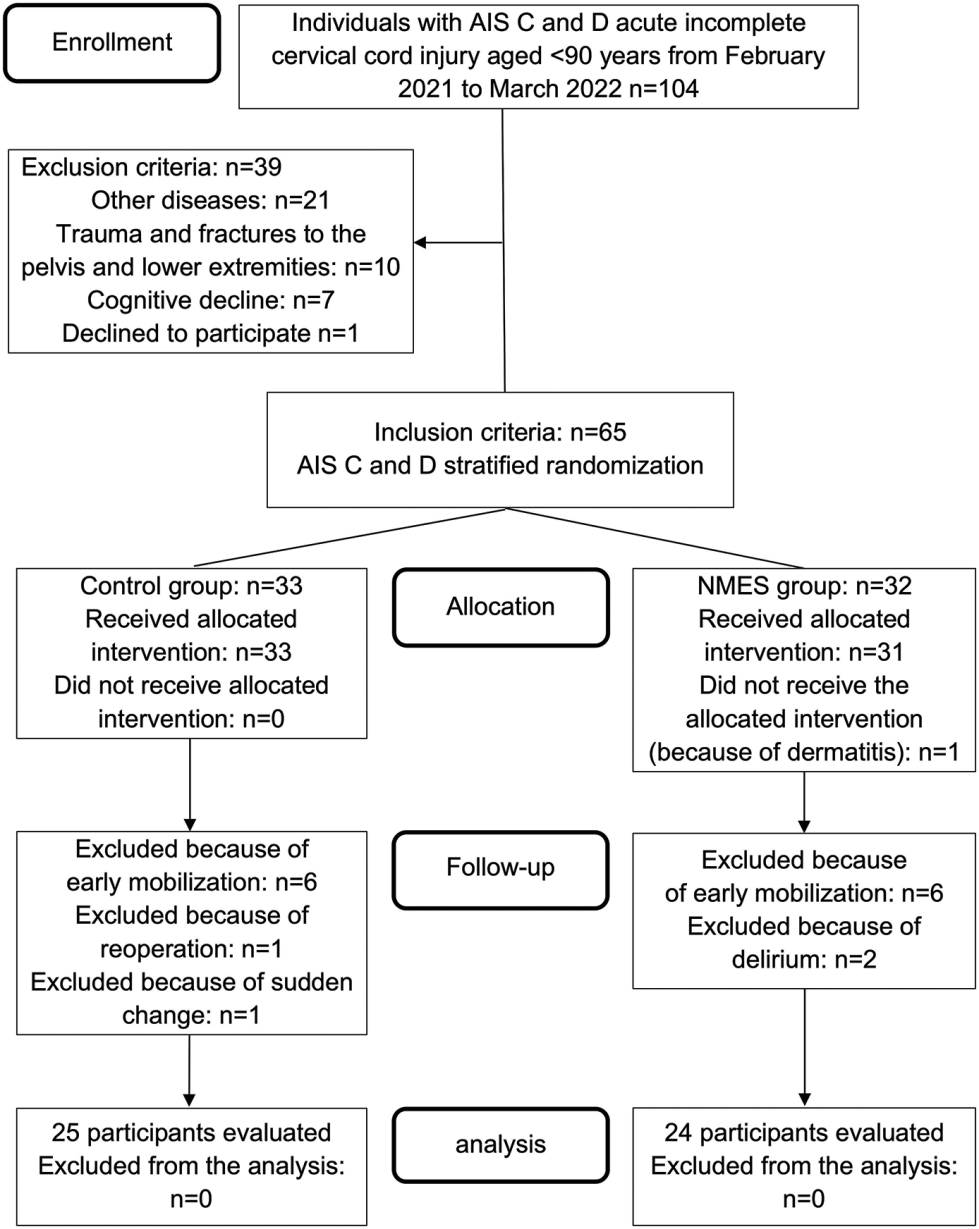
Study flowchart This flowchart illustrates the steps taken to select study participants. AIS, American Spinal Injury Association Impairment Scale; NMES, neuromuscular electrical stimulation

Of the 104 participants aged <90 years with AIS grades C and D who were admitted to the hospital within 48 h of injury due to SCI, 21 had diseases or disabilities affecting physical function, 10 had trauma or fractures in the pelvis or lower extremities, 7 had difficulties cooperating due to cognitive decline, and 1 declined to participate in this study. Sixty-five participants who met the inclusion criteria were stratified according to the AIS grade and were randomly assigned to the control (n = 33) or NMES (n = 32) group. During this study, 6 participants in the control group were discharged or transferred early, 1 underwent reoperation, and 1 had a sudden change in condition. In the NMES group, 6 participants were discharged or transferred early, and 2 developed delirium, all of whom were not followed up. One participant did not receive NMES after being assigned to the NMES group because of pre-existing dermatitis. However, the analysis was performed in the NMES group according to the modified ITT analysis. The final analysis included 25 and 24 participants in the control and NMES groups, respectively (Fig. 2).

No significant intergroup differences were found in any of the attributes (Table 1). The number of days from injury to the start of measurements was 3.4 ± 1.3 days for the control group and 2.7 ± 1.1 days for the NMES group, indicating a trend toward earlier measurements in the NMES group. Surgical treatment was performed on all patients, except 1 in the control group.

**Table 1.** Participant characteristics

	Control (n = 25)	NMES (n = 24)	*p*
Age (years)	67.7 ± 16.1	64.9 ± 12.6	0.51
Sex (male/female)	20/5	19/5	0.94
BMI (kg/m^2^)	23.5 ± 4.1	23.2 ± 2.8	0.80
AIS (C/D)	13/12	13/11	0.88
Injury mechanism			0.24
Fall on a level surface	11	12	
Fall	6	2	
Traffic accident	2	6	
Others	6	4	
Methods of treatment			0.33
Laminoplasty	18	21	
Fixation	6	3	
Conservative treatment	1	0	
Days from injury to start of PT (days)	1.7 ± 1.2	1.3 ± 1.2	0.22
Days from injury to start of measurement (days)	3.4 ± 1.3	2.7 ± 1.1	0.07
Initial serum albumin level (g/dL)	3.7 ± 0.5	3.7 ± 0.4	0.82
C-reactive protein (mg/L)	81.5 ± 12.9	76.7 ± 13.2	0.80

Values are presented as numbers or means ± standard deviations

NMES, neuromuscular electrical stimulation; BMI, body mass index; AIS, American Spinal Injury Association Impairment Scale; PT, physical therapy

### Changes in quadriceps muscle thickness

The quadriceps muscle thickness on the stronger and weaker sides changed from 32.04 ± 8.05 mm to 23.49 ± 6.80 mm and 30.56 ± 7.29 mm to 22.52 ± 6.77 mm, respectively, in the control group. The corresponding changes in the NMES group were from 30.74 ± 9.01 mm to 26.20 ± 7.81 mm and 28.84 ± 7.20 mm to 24.35 ± 7.22 mm, respectively, all of which were significant reductions (*p* <0.05). The relative differences between the initial and final results for the stronger and weaker sides were –25.7% ± 16.8% and –26.0% ± 13.3%, respectively, in the control group and –14.2% ± 11.3% and –15.1% ± 13.8%, respectively, in the NMES group, with significantly fewer reductions in both sides in the NMES group (*p* <0.05) (Table 2).

**Table 2.** Initial and final measurement (2nd week) of quadriceps muscle thickness and L3 motor score in the NMES and control groups

		Groups				Difference			
		Initial measurement		Final measurement		Control	NMES	*p*	95% CI
		Control	NMES	Control	NMES				
Muscle thickness	Stronger	32.04 ± 8.05	30.74 ± 9.01	23.49 ± 6.80	26.20 ± 7.81	–25.7 ± 16.8^*^	–14.2 ± 11.3^*^	<0.05	3.3–19.8
	Weaker	30.56 ± 7.29	28.84 ± 7.20	22.52 ± 6.77	24.35 ± 7.22	–26.0 ± 13.3^*^	–15.1 ± 13.8^*^	<0.05	3.1–18.7
L3 motor score	Stronger	4.0 ± 1.4	3.5 ± 1.6	4.4 ± 1.3	4.4 ± 1.2	0.4 ± 0.8	0.8 ± 1.2	0.16	–0.2 to 1.0
	Weaker	3.4 ± 1.7	2.7 ± 1.8	3.7 ± 1.6	4.0 ± 1.5	0.4 ± 0.8	1.3 ± 1.4	<0.05	0.3–1.6

Values are presented as numbers or means ± standard deviations

Final measurement: Measurements were made 2 weeks after the Initial measurement

*Relative difference: ([final – initial]/initial × 100)

NMES, neuromuscular electrical stimulation; CI, confidence interval

### Changes in L3 motor score

The scores for the stronger and weaker sides in the control group ranged from 4.0 ± 1.4 to 4.4 ± 1.3 and from 3.4 ± 1.7 to 3.7 ± 1.6, respectively. The corresponding scores in the NMES group ranged from 3.5 ± 1.6 to 4.4 ± 1.2 and from 2.7 ± 1.8 to 4.0 ± 1.5, all showing significant improvements (*p* <0.05). The initial and final changes for the stronger and weaker sides were 0.4 ± 0.8 and 0.4 ± 0.8, respectively, in the control group and 0.8 ± 1.2 and 1.3 ± 1.4, respectively, in the NMES group, showing significant improvement in the weaker side in the NMES group (*p* <0.05) (Table 2).

### Adverse events

No adverse events related to NMES, such as skin rash, myalgia, or discomfort, occurred in the NMES group during the procedure.

## Discussion

This study showed that early NMES within 2 weeks after injury could prevent muscle atrophy and significantly improve L3 motor score on the weaker side in hospitalized individuals with incomplete cervical SCI categorized under AIS grades C and D. In an early exercise intervention on muscle atrophy in individuals with SCI^16)^, three of the four randomized controlled trials showed the effectiveness of long-term NMES alone^17–19)^. These findings suggest the potential benefits of NMES for preventing muscle atrophy in subacute and complete SCI. However, further studies are required on its effectiveness in subacute and incomplete SCI. The average length of stay in acute care hospitals in Japan is approximately 2 weeks^29)^. Shorter hospitalizations for SCI make studying shorter interventions for muscle atrophy crucial. Several factors contribute to atrophy, including injury severity^30)^, inflammation^31)^, inactivity^16^^,^^32)^, aging^33)^, and impaired nerve function^31)^. Although active exercise can be challenging^16)^ owing to nervous system damage and complications^5^^,^^6^^,^^16)^, this study explored short-term NMES as a potential solution. All participants had traumatic injuries and underwent surgery, suggesting a postoperative systemic inflammatory response. This is further supported by elevated CRP levels, indicating systemic inflammation. Considering peak muscle loss in individuals in the intensive care unit within 2 weeks^13)^, this timeframe was selected for this study. Despite potential age-related limitations in recovery and atrophy risk in both groups, NMES significantly reduced quadriceps muscle thickness loss compared with control (14–15% vs. 26%). Therefore, short-term NMES can prevent muscle atrophy in subacute incomplete SCI.

Although NMES did not significantly improve the L3 motor score on the stronger side (with an initial measurement score of approximately 4), potentially due to a ceiling effect, it showed promising results on the weaker side (with an initial measurement score of approximately 3). This suggests a potential benefit of NMES for improving motor function in weaker limbs. This benefit might be linked to the relationship between muscle cross-sectional area and force generation^34)^, as demonstrated by Bochkezanian et al.^35)^ NMES significantly decreases the reduction in muscle thickness as a peripheral effect^36^^,^^37)^, resulting in the maintenance and improvement of muscle power output. In their review of the effects of NMES on muscle strength in individuals with SCI, de Freitas et al.^32)^ were inconclusive on the effects of NMES on motor scores owing to limitations in sample size and inconsistency in measurement methods. Our study addressed these limitations by employing a randomized controlled trial with a calculated sample size and using the internationally recognized L3 motor score as the outcome. However, L3 of the lower extremity motor score is an ordinal scale and may not be sensitive to changes in motor score. In addition, although our study addressed the limitations noted by de Freitas et al.^32)^ using a randomized controlled trial design and a carefully calculated sample size, the final sample size was slightly below the target. This minor shortfall may slightly affect our results’ statistical power and precision. Nevertheless, the significant improvements observed in muscle atrophy prevention and the L3 motor score on the weaker side provide evidence for the potential benefits of NMES. Considering the sample size, these findings should be interpreted cautiously.

The short-term treatment outcomes of this study showed that preventing muscle atrophy could offer advantages in force generation^35)^, and improving motor scores may positively influence gait prognosis^38)^. A recent study in patients with subacute incomplete SCI reported that changes in rectus femoris muscle thickness significantly correlated with walking ability and activities of daily living (ADL) at 1 year^39)^. This result supports our consideration.

The NMES settings were determined by previous studies^22^^,^^24)^, and the stimulus intensity was set to an intensity acceptable to the participants to avoid pain and discomfort. No NMES-related adverse events, such as myalgia, discomfort during exercise, or skin rash, occurred, and the NMES group participants did not drop out for these reasons. Therefore, NMES can be safely performed in individuals with subacute incomplete cervical SCI.

A limitation of this study is that although we investigated quadriceps muscle thickness and L3 motor score as indicators of NMES effectiveness in individuals with subacute incomplete cervical SCI, we could not assess its effects on the most crucial outcomes and improvements in walking ability and ADL. Second, the results may have been influenced by the fact that the participants and their physiotherapists needed to be adequately blinded. The control group could also have been blinded by performing a sham NMES^40)^. In addition, as the hospital was an acute care hospital, several participants underwent early transfer after completing initial treatment (such as surgery) according to the participant’s or family member’s wishes or the protocol in the hospital ward. Thus, the resultant dropouts may have caused bias. Therefore, an actual ITT analysis was difficult, and a modified ITT analysis was used. However, as the number of participants who dropped out was the same in both groups, the effect of this factor on the final analysis results was limited. Future studies should address these limitations and evaluate long-term functional outcomes.

## Conclusion

In summary, in this study, we investigated the effects of NMES on muscle atrophy and L3 motor score in individuals with subacute incomplete cervical SCI. The results showed that compared with PT alone, NMES combined with PT reduced quadriceps muscle atrophy and improved the L3 motor score values on the weaker side.

## Acknowledgments

The authors would like to thank Kenta Togashi, Ayaka Kawase, Shinta Araki, and Ikumi Suzuki of Saitama Medical Center for their cooperation in the recruitment process, especially the participants of the study.

## Funding

This study was supported by the SMU-FHMC (grant number 14-024).

## Conflicts of Interest

The authors declare that there is no conflicts of interest.
